# Indents

**Published:** 1906-01-15

**Authors:** 


### INDENTS.

**☞ Brief Articles of Technical or Scientific Value will receive notice and comment. Contributions invited.**

**Setting Plaster.** After the plaster is mixed scrape into it from an old plaster, model a little plaster to facilitate the setting.

**Sterilizing Mouth Mirrors.** Place the mirrors in strong alcohol until needed for use. Then wash in clean water and dry with a sterile napkin.—E. M. Kaptan, in *Review.*

**Stuttering Cured by Correcting Malocclusion.** Dr. L. P. Bethel, of Columbus, claims to have cured a child from stuttering by correcting a class II protrusion case. The intermaxillary rubber bands were used.—*Dentist's Magazine.*

**Broach Sterilization.** J. Leon Williams in the *Cosmos,* says to sterilize a broach, hold it a little above the flame of an alcohol lamp to avoid burning it, until it is thoroughly hot, then withdraw gradually away from the flame to avoid hardening it by a too sudden chilling in the cold air.

**Adaptation of Partial Dentures.** When only one or two teeth remain, as the two upper canines for instance, a closer adaptation may be secured by slightly trimming the plaster teeth and completely encircling them with soft or velum rubber. Pack the ordinary rubber around this and vulcanize as usual. This will give a support superior to that given by clasps and less harmful to tooth structure.—*International Dental Journal.*

**A Good Coating for Plaster Casts.** To four ounces of sulphuric ether add two ounces of collodion and two ounces of "silver gloss." The latter may be obtained from dealers in painters' supplies and is put up in one-ounce packages. Let the mixture stand for about forty-eight hours, and shake well before using. Apply with a camel-hair brush and keep in well-corked bottle. This will give a beautiful glossy surface to casts.—Dr. J. F. Steele, in *American Journal.*

**Sharpen a Lathe Cone.** If the thread of the cone on your lathe has worn so smooth that it will not hold your brush wheels or polishing wheels, take a small three-cornered file and hold the edge in the grooves of the thread and run the lathe toward you. Allow your arms to carry the file at the same angle as it moves up on the cone. By chasing over the cone a few times you will be pleased to find that you have a new sharp thread that will hold the wheels steadily.—C. F. Graham, in *Dominion Journal.*

**Cleaning Teeth.** Cleaning teeth for show and cleaning teeth conscientiously for the preservation of their health are two different things. The patrons of our profession, however, do not always appreciate the difference, especially when time must be consumed to do honest work and a fee charged accordingly. Some dentists—too many have a fixed fee, and a small one at that, for cleaning teeth, with the result that only the effort is expended that in their estimation fits the fee, and that is mostly mere cleaning for show. One cannot prevent pyorrhea in that way.—R. B. Tuller, in *American Dental Journal.*

**Perfect Contact for High Pressure Needle.** With a No. 6 round bur ream out the soft decay to a saucer-shaped cavity. Then use No. 66½ "Revelation" fissure bur to drill a pit sufficiently deep to securely embrace the point, and to penetrate to the sensitive dentine. With a cone or No. 45 bud-shaped bur ream the outer orifice of the pit to fit the bevel of the needle. The location of the pit should be within the cavity wall when practicable so that it will be covered by the filling. It can be made anywhere, however, and afterward filled.—Dr. Weaver, in *Dentists' Magazine.*

**Hot Air as a Pyorrhea Treatment.** In the discussion of a paper read before the Chicago Odontological Society and printed in the January *Dental Review,* Dr. George W. Cook suggests that hot air injected into Pyorrhea pockets should promote healing. Dr. Brophy states that he has used it advantageously after curretting the antrum. He thinks its virtue in this case depends on the fact that it dries the surface and produces increased absorption of remedies applied as antiseptics or stimulants. He therefore thinks the drying of the pockets previous to medication should materially assist the penetration of the medicaments.

**Silver Nitrate with Cement Fillings.** A cement filling, oxyphosphate of zinc, placed upon a surface treated with nitrate of silver, will, for some reason, last a great deal longer and be a great deal better mass than the same mass not having the peculiar effect it gets from this film of silver albuminate. A great many, no doubt, have seen the effect of nitrate of silver upon a surface which has been infected to a very slight depth. That in time will become a polished black surface and further decay will never result. Now, if in deeper cavities we can make partial preparation and apply nitrate of silver, and have it last longer than otherwise, it is worth knowing.—W. V.-B. Ames, in *Review.*

**Irish.** The following is taken from a report in one of our exchanges of a discussion that took place in a dental society meeting, and illustrates how careless we often are in talking and how negligent an editor may be. The gentleman was discussing the comparattive values of inlays and gold fillings, and his point was to show what a superior operation he had made by cementing into the cavity a filling which he had accidentally removed from the cavity while finishing with the burnisher, and comparing it with one made in an adjacent tooth at the same sitting and which did not come out during the operation of finishing, but which eventually turned dark around its margins. He says: "The one tooth is black as can be around it (meaning the filling), *but the one I put in and took out is in there yet and doing good service.*

**Putrescent Pulps in Deciduous Teeth.** Where you are able to place a root filling such as was formerly deemed proper in these cases, put in a dressing of medicinal agents that contain formaldehyde, which will act as a preservative, is easily placed. This will do away with so much fatigue that little patients experience with long sittings. I not only practice this in many cases of deciduous teeth, but in some cases of first permanent molars. This is not intended for permanent work, but it will preserve the tooth, retain it in the mouth and give the patient the use of it until he has arrived at an age where his reason can be appealed to so that a permanent filling may be inserted, as it should be done.—B. C. Campbell, in *Review.*

**Pain after Extraction.** Thoroughly currett the alveolus, thus removing all disorganized tissue, coagulated blood, and alveolar debris, if any is present. The alveolus is then copiously irrigated with hot water, and after drying with cotton, an application is made of two drops of pure carbolic acid, and the alveolus is loosely packed with sterilized gauze. If the pain does not subside, an application should be made of campho-phenique and morphin-acetate. A small pellet of cotton is saturated with campho-phenique, and after taking up with it a small amount of morphin-acetate, is carried to the bottom of the painful alveolus.

The morphin-campho-phenique dressing produces most remarkable results in the sense that the pain disappears almost instantaneously with the introduction of the dressing. —Dr. Endelmann.

**Is a Painless Dental Operation Possible?** With some operators it is already an accomplished fact as their patients so strongly testify; with others it never will be. So far as the patients themselves are concerned it is not possible, they cannot bring it about as much as they would like to do. But from the dentist's standpoint it is possible, but only for him who cares enough for its accomplishment to be determined, at any trouble and cost, to give it to his patients. He must be determined and able to hold himself to his task with an earnestness and strength that most think they have got but do not prove themselves to have. A strength that will enable him not only to give up an older method for a better, but a strength that will make or in some way obtain a better when the old one is painful. He will not come to it in one bound or by one mighty effort, but by a continuous mighty effort watching and studying every detail with this goal in view.—Dr. W. A. Price, in *Dentist's Magazine.*

**High Pressure Anesthesia, Points to Remember.** *First.* See that the injector you are using does not leak, and,

*Second:* That the tapered point or nozzle is in good condition.

*Third:* See that aperture of drill-hole in tooth is perfectly smooth.

*Fourth:* Do not employ too strong cocain solutions, 5 percent.

*Fifth:* Be sure drill-hole reaches the dentine.

*Sixth:* In removing pulps, after pulp cavity has been reached, proceed gently and with great caution.

*Seventh:* If you do not succeed the first or second time, try again, as this method once mastered will prove a great boon to yourselves as well as to your patients.

*Eighth:* This method, when mastered, will enable one to get satisfactory results in every case.—Dr. C. G. Meyers, in *Dentist's Magazine.*

**Sterilization of Cutting Instruments.** Of fifty prominent surgeons who were asked their practice in the sterilization of knives and scissors by H. A. Royster (*Annals of Gynecology and Pediatrics*, February 1905), twenty boil them, two use dry heat and twenty-one use chemical agents, chiefly carbolic acid, alcohol or both. Many complaints are made of the ruining of cutting instruments by the process of sterilization. The author has made experiments to determine the effects of the various methods, and arrives at the following conclusions: (1) Knives can be safely sterilized by chemical and mechanical means without the use of heat in any form. (2) The majority of American surgeons are using carbolic acid or alcohol, or both. (3) Immersion in 95 percent alcohol has the least and boiling the most effect in dulling the edge of a knife.—*Detroit Medical Journal.*

At St. Winifred's Hospital, San Francisco, Dr. Winslow Anderson uses 5 percent formalin in absolute alcohol for all cutting instruments.—*Pacific Medical Journal.*

**Sodium Dioxid Bleaching.** The dam is placed over the tooth and adjacent teeth. A thin platinum band is wrapped around the tooth to be bleached and white gutta-percha warmed and used to form a pocket about the cavity.

By the use of a small gold or platinum spoon, some sodium dioxide is placed in the cavity and forced some distance up the root canal with a glass instrument. Distilled water is now dropped into the cavity and the band of platinum held over the cavity to force the generated oxygen into the dentine. After sufficient time to allow the oxygen to work the cavity should be washed and dried, and the operation repeated if necessary.

Should it be found impossible to remove the pigment mechanically with water, a 3 percent solution of sulphuric acid may be used to chemically dissolve it, after which wash with water and let dry, preferably without using hot air. Now burnish a paste to precipitate of calcium phosphate and distilled water into the lower third of the root and against all exposed dentine. Make a base for final fillng, using light colored cement.—R. B. Tuller, in *American Journal.*

**Duplication of Plaster Models.** To one teacup of water add a half pound of French gelatine, heat until the liquid will drip readily from a stirring rod, cool until the hand can be dipped in it and then pour around the model and allow to stand over night. The model is previously prepared by simply dusting over thoroughly with powdered soapstone and then placing it in a mold to limit the shape of the gelatine. The next day remove gelatine mold from model and coat with a saturated solution of alum water. When dry, brush over this a solution of 2 pwt. melted stearine and one ounce kerosene. When dry dust lightly with powdered soapstone, fit into the containing mold and pour in the plaster of Paris. Care must be taken to separate mold from plaster as soon as plaster sets and *before heat becomes great enough* to melt the gelatine. This mold may be used repeatedly until it gets too hard to be separated, when it may be remelted and used again. The alum solution hardens the sufrace and the stearine solution and soapstone provide easy separation. Models may be coated with a varnish made by dissolving white wax in spirits of turpentine.—Dr. W. H. Ellis, *Dentist's Magazine.*

**Amalgam.** Dr. J. D. Patterson, in the *Kansas City Dental Journal,* gives the following rules for the use and manipulation of amalgam:

*Rule First.* Use gold filling, gold inlays or porcelain inlays instead of amalgam whenever an equally durable operation can be made.

*Rule Second.* In using amalgam, select a standard ternary or quaternary compound; alloys of silver and tin alone, in whatever proportion, are found to be unreliable.

*Rule Third.* Weigh the alloy and mercury, using an amount of mercury that will make a homogeneous mass when placed in the cavity under strong pressure, without pressing out a surplus of mercury. This proportion is usually one of mercury to three of the alloy by weight. For convenience use a Fletcher balance in weighing.

*Rule Fourth.* Mix the alloy and mercury by shaking in a mixing-tube or test-tube. This gives a more perfect and even mix than can be made by other methods, and is also rapidly done. Make into ingots with a Fletcher ingot mold for convenience in handling.

*Rule Fifth.* Make all cavities into simple ones with the most suitable matrix, and in placing the filling use a small portion, or one ingot, at a time, building it together with hard hand-pressure and with a mallet. The formation of a cavity should be upon the same lines as for gold, except that the thin edge, sometimes admissible, with gold, is a source of weakness with the alloy.—*Kansas City Dental Journal.*

**Dentists and the Societies.** With such a record of influence and accomplishment, to which every dentist is more or less indebted, it sometimes seems odd that our societies, generally speaking, are not of greater influence, and more enthusiastic and larger membership; but when we stop to consider the cosmopolitan character of the dental profession, and realize what a comparatively small proportion of it is composed of studious, thoughtful, well educated men, there is no longer cause for surprise at the condition of our societies. We feel, however, that remedies may be applied which will in time produce results most beneficial to all concerned. Raising the standard for entrance into and graduation from our dental colleges will have its effect, and in time will bring into our profession a large proportion of members fitted by early education to take an active part in its literary and scientific progress.

Our societies themselves will in the course of time, and as a result of manifold experiences, find means to encourage their willing and energetic members, and to eliminate the drones and idlers who now unfortunately comprise so large a precentage of their membership.

Encouragement might be extended to writers or clinicians by making the offices at the disposal of the Society stand for honor and reward conferred on those who for a year had done most to advance the interests of the society and interest in its meetings. Membership might be restricted to those who, aside from being reputable and ethical practitioners, promise in their applications for membership to contribute to the program of the various sessions at least once each year, a still greater improvement in the affairs of our societies will be manifest whenever our colleges include in their course of study a thorough training in the history, literature and ethics of dentistry.—Dr. Platt, in *Pacific Gazette*.

**Is the Painless Dental Operation Profitable.** From the patients standpoint painless operations may mean larger fees to pay, but not in such proportion as to make them less desirable. From the patient's standpoint they may contribute to good health, more comfort, more nerve strength, more happiness and more durable work.

From the dentist's standpoint it will be so profitable in every way that almost without exception he would leave the practice of dentistry rather than return to his former more painful methods. If to him *profitable* means more patients, it certainly will bring them, for everywhere, everybody, himself included, is looking and planing to avoid pain, the greatest menace to happiness. If *profitable* means more reward in fees for his service, a very legitimate ambition for worthy service, he certainly will get it, for none pay so cheerfully and liberally as appreciative patients, and most people would gladly pay more not to be hurt than they would for the operation itself. If *profitable* means to him a consciousness of having done for humanity a great good, a worthy reward for a worthy man, he will have a boundless joy in his professional life that the narrow, selfish operator never has dreamed possible. From the dentist's standpoint it is certainly profitable, no matter from what way he views it, and will bring him returns in many times larger proportion for the outlay than any other special efforts he can make.

*Is it right?* How many men think the world owes them a living when it does not owe them anything until they earn it, but is ready to pay them promptly and liberally when they do.

All social laws are based on the promises that we have a duty each to the other as well as to himself. No man has a right to take from another, simply for profit, his life or any part of his life forces and under no circumstances more of them than is necessary for his, the patient's best good, and not the operator's.

No man has a right to practice dentistry and take from his patient more of his life force than is necessary, he is a despoiler and thief among society who does so.

There is no higher trust, except life itself, than that given to the members of the dental profession, and the only reason *public sentiment,* the great social administrator, does not brand and expel from our profession, as despoilers and traitors to their commission, a large part of its devotees, is because she is in darkness and ignorant of the real facts that very many of us know so well. There is no greater right and duty to come to any of our lives than the duty to give to our fellow men the best service which is in dentistry the least pain with best results.—Dr. W. A. Price, in *Dentist's Magazine.*

